# Focal Myocarditis As the First Sign in the Presentation of a COVID-19 Infection: A Case Report

**DOI:** 10.7759/cureus.26358

**Published:** 2022-06-27

**Authors:** Mohamad Zayour, Rana Al Ashkar, Mahmoud Karaki, Elie Chammas, Wassim Shatila

**Affiliations:** 1 Cardiology, University of Balamand, Beirut, LBN; 2 Internal Medicine, Faculty of Medicine, Lebanese University, Beirut, LBN; 3 Diabetes and Endocrinology, University of Balamand, Beirut, LBN; 4 Cardiology, Clemenceau Medical Center, Beirut, LBN

**Keywords:** atypical presentation, clinical case report, covid 19, sars-cov-2, covid and myocarditis

## Abstract

Coronavirus disease 2019 (COVID-19) is caused by severe acute respiratory syndrome coronavirus 2 (SARS-COV-2). Patients with COVID-19 typically present with symptoms and signs related to respiratory tract infection. However, a broad spectrum of cardiac manifestations including myocarditis has been reported as complications of this virus. Nevertheless, focal myocarditis as the first clinical manifestation of COVID-19 infection has not been reported before.

Thus, we herein present the case of a 56-year-old male patient previously healthy and presented to the emergency department with chest pain. The clinical picture was compatible with inferior ST-elevation myocardial infarction (STEMI). Initial COVID-19 polymerase chain reaction (PCR) was negative, as well for its classic symptoms. Thereafter, further investigations suggested the diagnosis of focal myocarditis. Later on, the patient started to have a fever and repeated COVID-19 PCR that returned positive.

## Introduction

Patients with COVID-19 typically present with symptoms and signs related to respiratory tract infection [1]. Moreover, cardiac manifestations and complications effects of the illness were reported ranging from myocardial injury, heart failure (HF), cardiogenic shock, and cardiac arrhythmias to sudden cardiac arrest. COVID-19-related myocarditis cases have been described in the literature with a variable range of clinical presentations [2-5].

## Case presentation

A 56-year-old male patient, a heavy smoker, previously healthy, presented to the hospital for sudden onset of oppressive chest pain radiating to the left shoulder and arm without any other accompanying symptoms. History is relevant for double dose COVID-19 vaccination (Pfizer biotech) with the second shot received around six months ago.

Upon Emergency Department presentation, the patient was hemodynamically stable with no fever, tachycardia, hypotension, or desaturation. Initial laboratory tests, presented in Table 1, were normal. Troponin level majorly increased the next day.

**Table 1:** Table demonstrating the initial Lab values and repeat troponin

Lab	Value	Comments
Hemoglobin	14.8 mg/dL	
WBCs	12.1 x10^3 /ul	
Neutrophils	52.3%	
ESR	10 mm/Hr	
CRP	0.562 mg/dl	
Creatinine	0.89	
Initial troponin (admission day 1)	<0.01 ng/ml	undetectable
COVID-19 PCR	negative	
Troponin	16.55 ng/ml	at day 2

**Figure 1:**
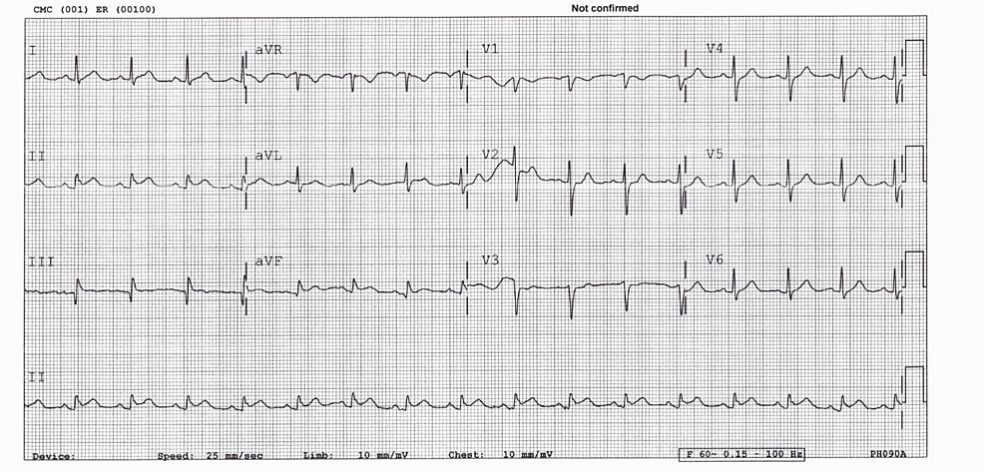
ECG showing ST-segment elevation of around 1 mm in the inferior leading suggestive of inferior STEMI.

**Figure 2:**
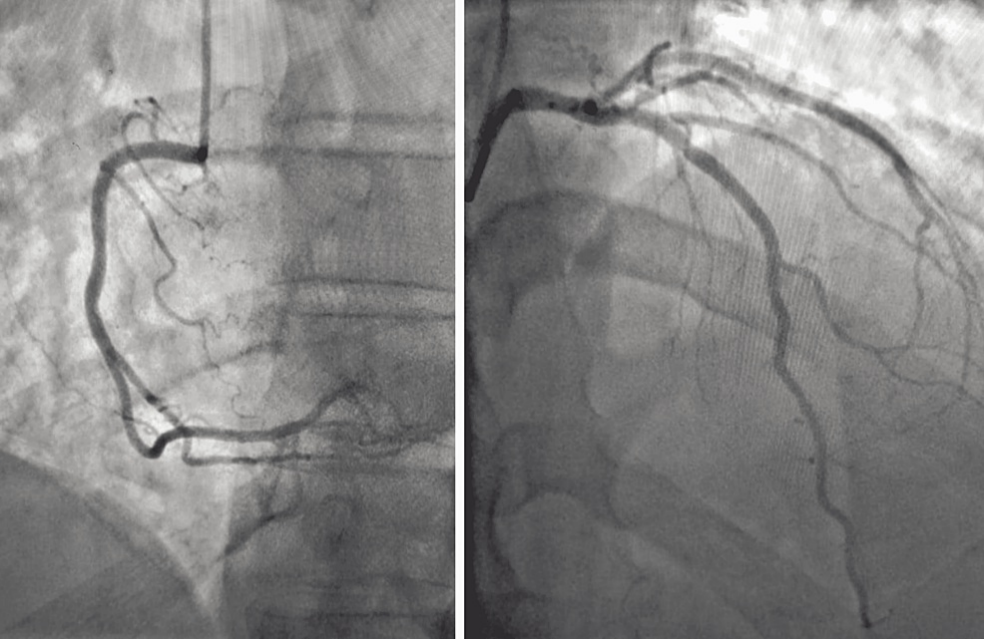
Coronary angiography showing no evidence of obstructive coronary artery disease.

**Figure 3:**
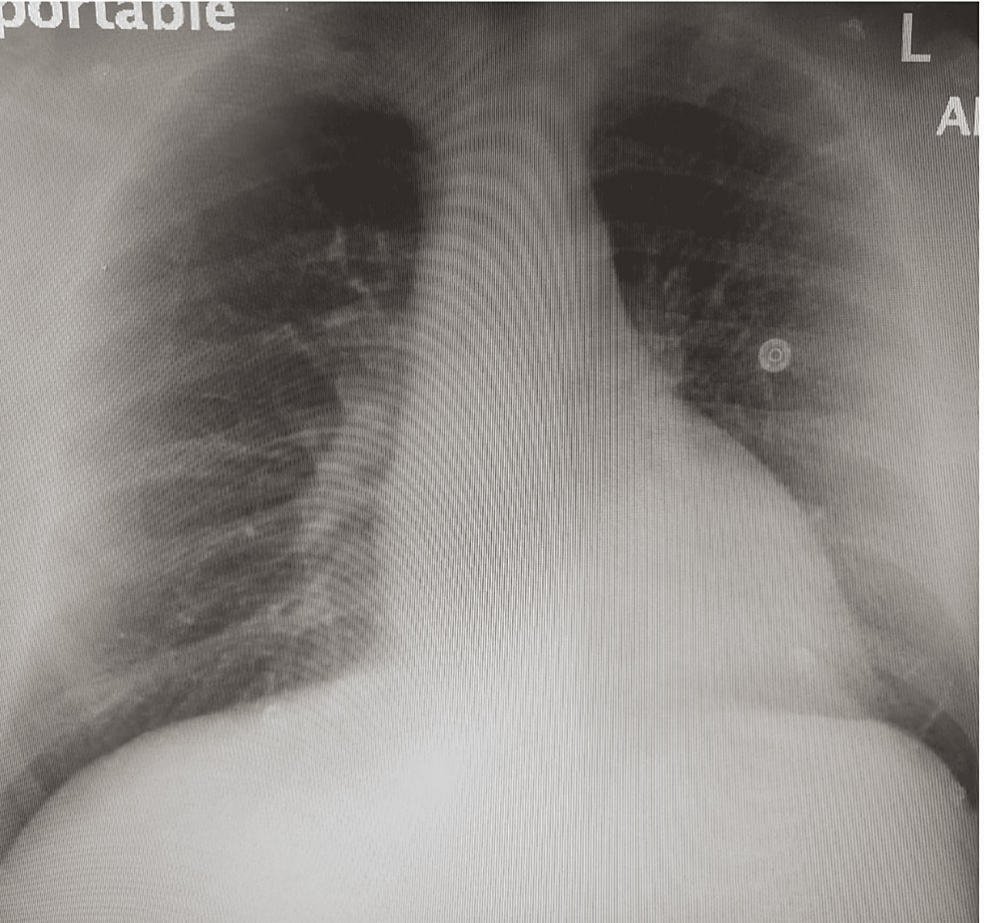
Normal portable chest x-ray.

An electrocardiogram (ECG) showed ST-segment elevation of around 1 mm in inferior leads (Figure 1). Thus, the diagnosis of possible inferior STEMI was considered. Consequently, the patient was started on acute coronary syndrome (ACS) treatment and underwent urgent cardiac catheterization that showed no evidence of obstructive coronary artery disease (Figure 2). A chest x-ray (CXR) done as a routine procedure showed no major abnormalities, note that the CXR is of poor quality (Figure 3).

**Figure 4:**
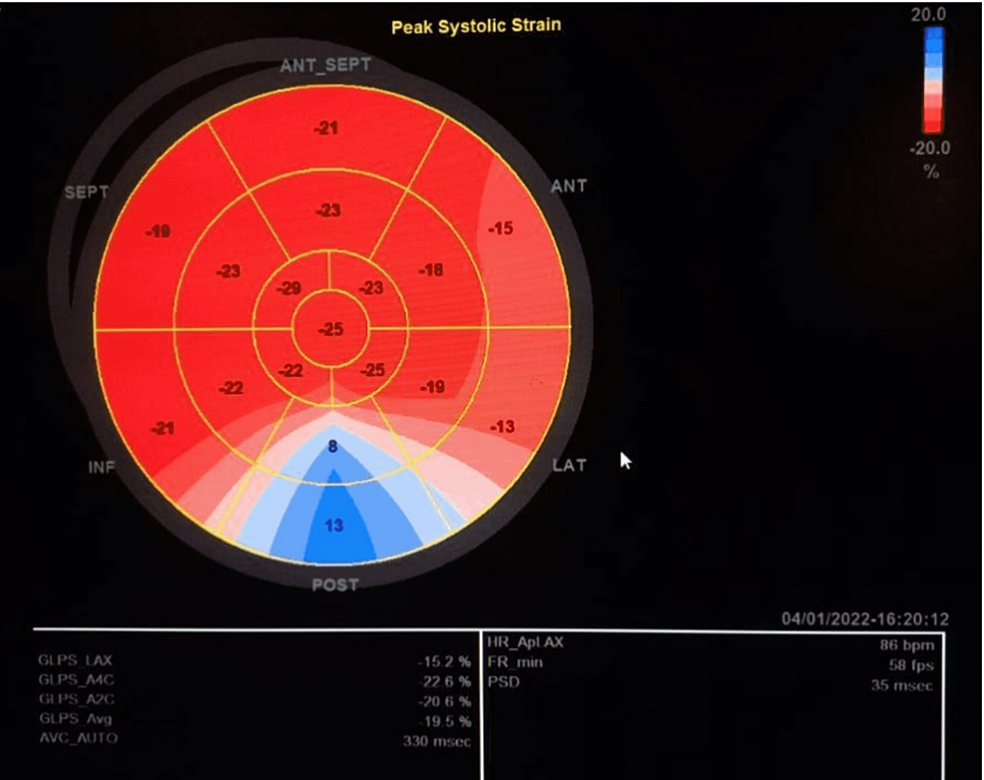
Echocardiography showing decrease in longitudinal strain mainly in posterior segment.

**Figure 5:**
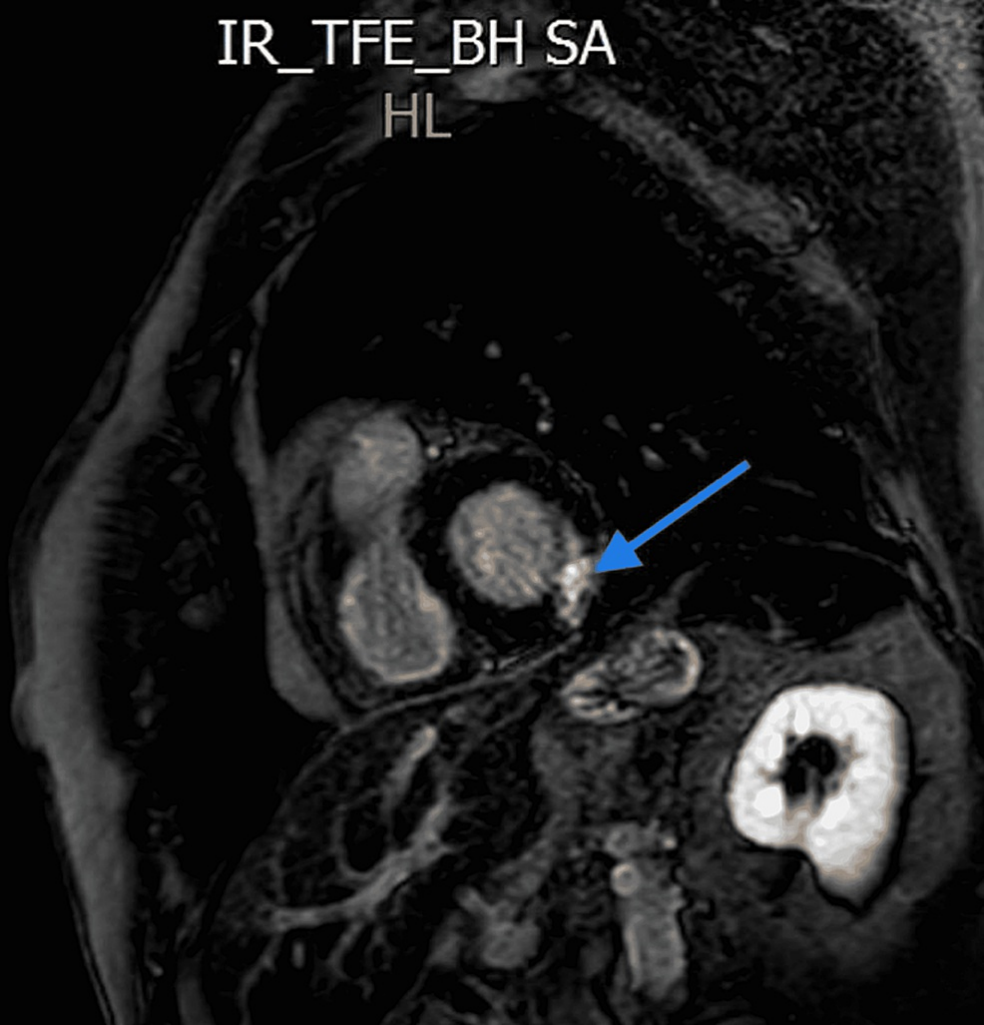
MRI T2 sequence showing mid myocardial increased signal in the inferolateral wall suggesting myocarditis (blue arrow).

**Figure 6:**
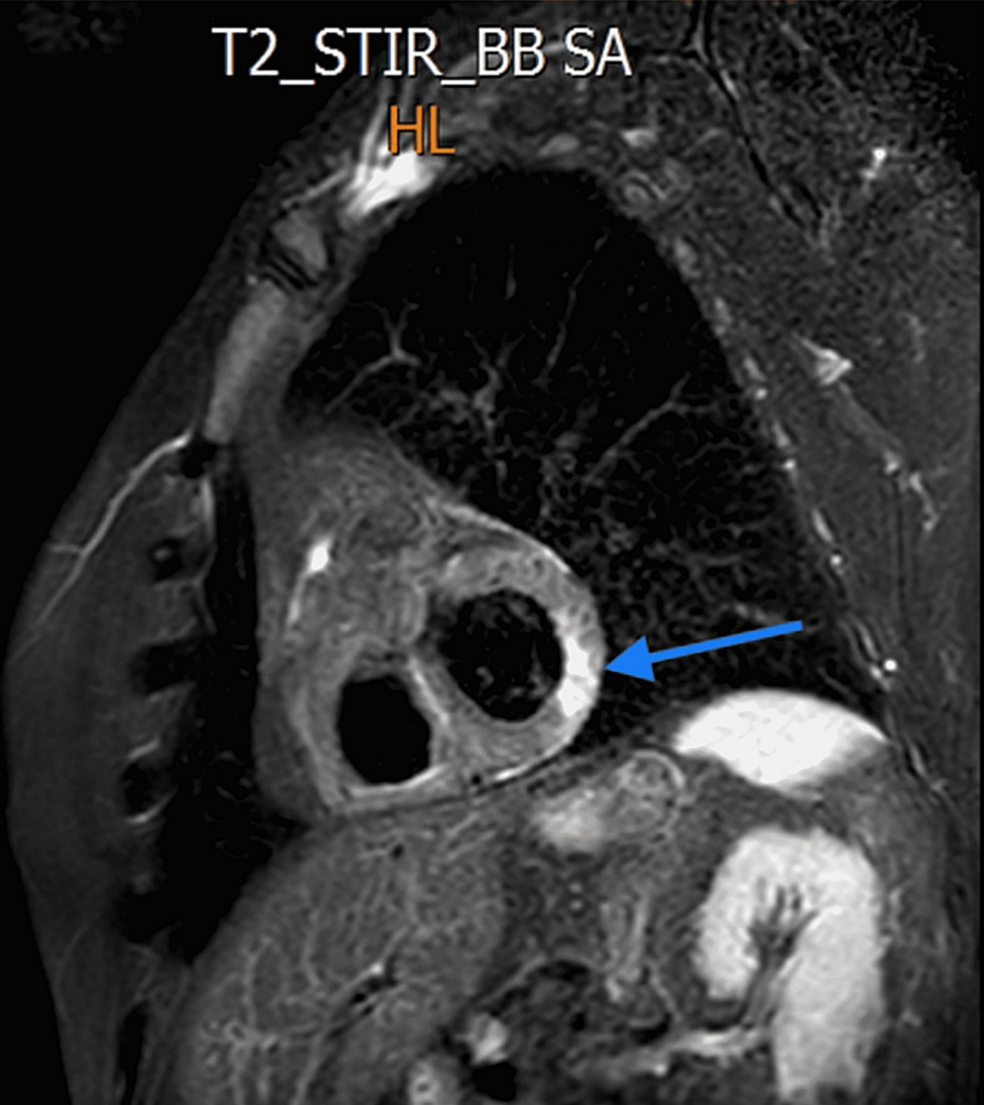
MRI late gadolinium enhancement uptake showing mid myocardial scar in inferolateral wall (blue arrow).

An echocardiogram revealed good contractility with no visible wall motion abnormalities but showed a decrease in longitudinal strain mainly in the posterior segment (Figure 4). The patient was discharged on Aspirin and Colchicine. Further investigations (done as out after two days) included cardiac MRI which revealed normal global LV and RV systolic functions with no wall motion abnormalities but the hyperintense signal at the level of the inferolateral segment with late gadolinium enhancement uptake fulfilling the criteria of focal myocarditis (Figures 5, 6). These findings are compatible with acute myocarditis involving the inferolateral wall. 

A few days after discharge, the patient started to have a fever along with generalized fatigue without any respiratory complaints. Repeated COVID-19 PCR returned positive. Medication was stopped after the patient’s symptoms subsides. 

## Discussion

Myocarditis is defined as an inflammatory disease of the myocardium, attributed to multiple infectious and non-infectious etiologies. It can be acute, subacute, chronic, focal, or diffuse, with variable clinical presentation ranging from mild symptoms of chest pain and palpitations associated with transient ECG changes to life-threatening cardiogenic shock and ventricular arrhythmia [2-4,6]. In all cases of suspected myocarditis, it is mandatory to exclude coronary artery disease, as well as other cardiac and noncardiac conditions. Furthermore, myocarditis and accompanying complications are associated with an important mortality rate, requiring appropriate diagnosis and management.

SARS-CoV-2 appeared to have a wide range of manifestations on the cardiac system with an idiopathic mechanism to date [5]. Some studies suggest that the expression of angiotensin-converting enzyme 2 (ACE2) receptors of SARS-CoV-2 in cardiac myocytes is responsible for the relatively high cardiovascular involvement in COVID-19 [7,8]. Human coronavirus-associated myocarditis is well known, and several SARS-CoV-2-related myocarditis cases have been reported in the literature during the COVID-19 pandemic [5]. Furthermore, the described myocardial injuries occur mostly during the course, as a complication, or even after this viral infection [2-4]. However, the occurrence of focal myocarditis as the first manifestation of COVID-19 three days before developing the classical symptoms, yet with an initial negative polymerase chain reaction (PCR), has not been described previously. The pathophysiology behind myocardial injury is multifactorial including direct viral toxicity as well as host response manifested by a cascade of inflammatory cytokines and immune cells resulting in myocyte death, even without respiratory involvement [9,10].

Regarding the clinical presentation of COVID-19-related myocarditis, it is variable among patients, ranging from mild symptoms (dyspnea, fatigue), to chest pain and tightness upon exertion [2], reaching tachyarrhythmias with acute onset HF and cardiogenic shock [5]. The diagnostic approach for patients with suspected COVID-19-associated myocarditis is similar to any cause of myocarditis along with positive PCR. Usually, laboratory tests including inflammatory markers C-reactive protein (CRP), erythrocyte sedimentation rate (ESR) [1], and procalcitonin are elevated in keeping with ongoing inflammation; however, normal values do not exclude myocarditis. Similarly, cardiac enzymes and N-terminal pro-B-type natriuretic peptide (NT-proBNP) may be elevated secondary to myocardial injury and ventricular dilation [2-4,6]. Moreover, ECG abnormalities are commonly seen in myocarditis such as ST-segment elevation or depression, T-wave inversion, sinus tachycardia, new-onset bundle branch block, and others [6], yet their absence is not exclusionary. Also, non-invasive imaging techniques such as cardiac magnetic resonance (CMR) imaging are essential in making the diagnosis of myocarditis and monitoring its progression. As well, an endomyocardial biopsy may be performed when the diagnosis was unclear [6,11]. Similarly, one should pay attention to the importance of longitudinal strain in the diagnosis and prognosis of myocarditis [12,13]. While no specific guidelines are available on the management of COVID-19-associated myocarditis, the optimal approach for myocardial injury is largely similar to other myocarditis etiologies [11].

In our case, the patient’s clinical presentation was compatible with ACS. But as coronary angiography was negative and since EKG changes were present along with detectable troponin and abnormal longitudinal strain, the likelihood of myocarditis was increased, hence confirmed by MRI. Nevertheless, because PCR-proven COVID-19 infection developed several days after discharge, his myocarditis was attributed most likely to this viral infection. Thereafter, the patient was discharged on aspirin and colchicine with subsequent great improvement of his symptoms during his follow-up appointment.

## Conclusions

This article described the case of an elderly patient with chest pain as the main presenting complaint. All the labs and imaging were indicative of focal myocarditis. The only positive clinical correlation for this presentation was a previous COVID-19 infection. Thus, COVID-19 cardiovascular manifestation should be always kept in mind in the differential diagnosis of patients presenting with typical cardiac symptoms even in the absence of the classic picture of COVID-19 infection.
